# Supplementary figures and images for: Dissecting surveying behavior of reactive microglia under chronic neurodegeneration
Source: eLife. 2026 Jan 8;14:RP107650. doi: 10.7554/eLife.107650 (PMC12782555; doi:10.7554/eLife.107650)

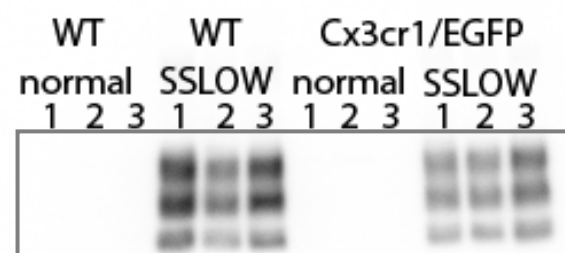

Supplement: Figure 1—figure supplement 1—source data 1. [file elife-107650-fig1-figsupp1-data1.zip › FigureS1d-labeled.pdf]

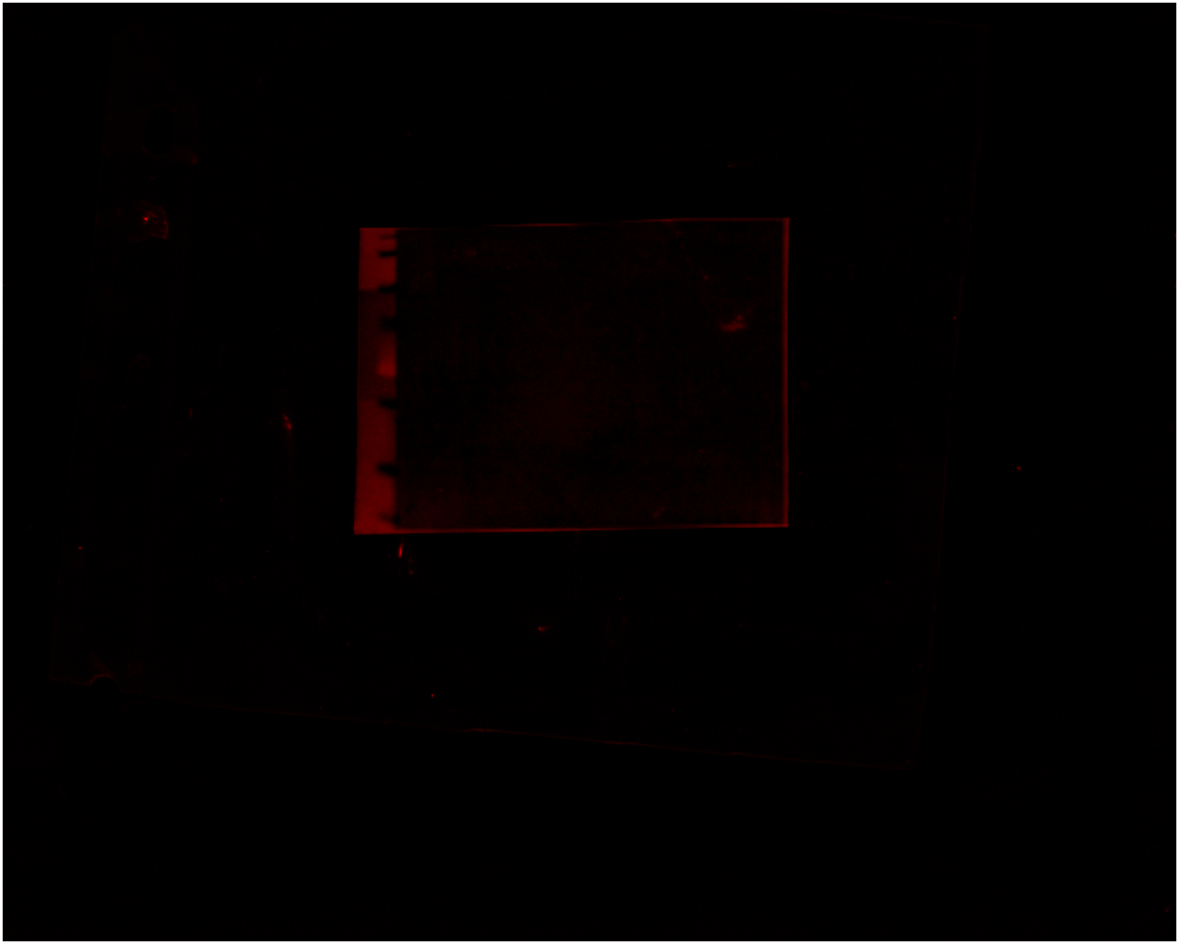

Supplement: Figure 1—figure supplement 1—source data 2. [file elife-107650-fig1-figsupp1-data2.zip › FigureS1d-original/UNIVERSAL_04112025_123514AlexaFluor546.tif]

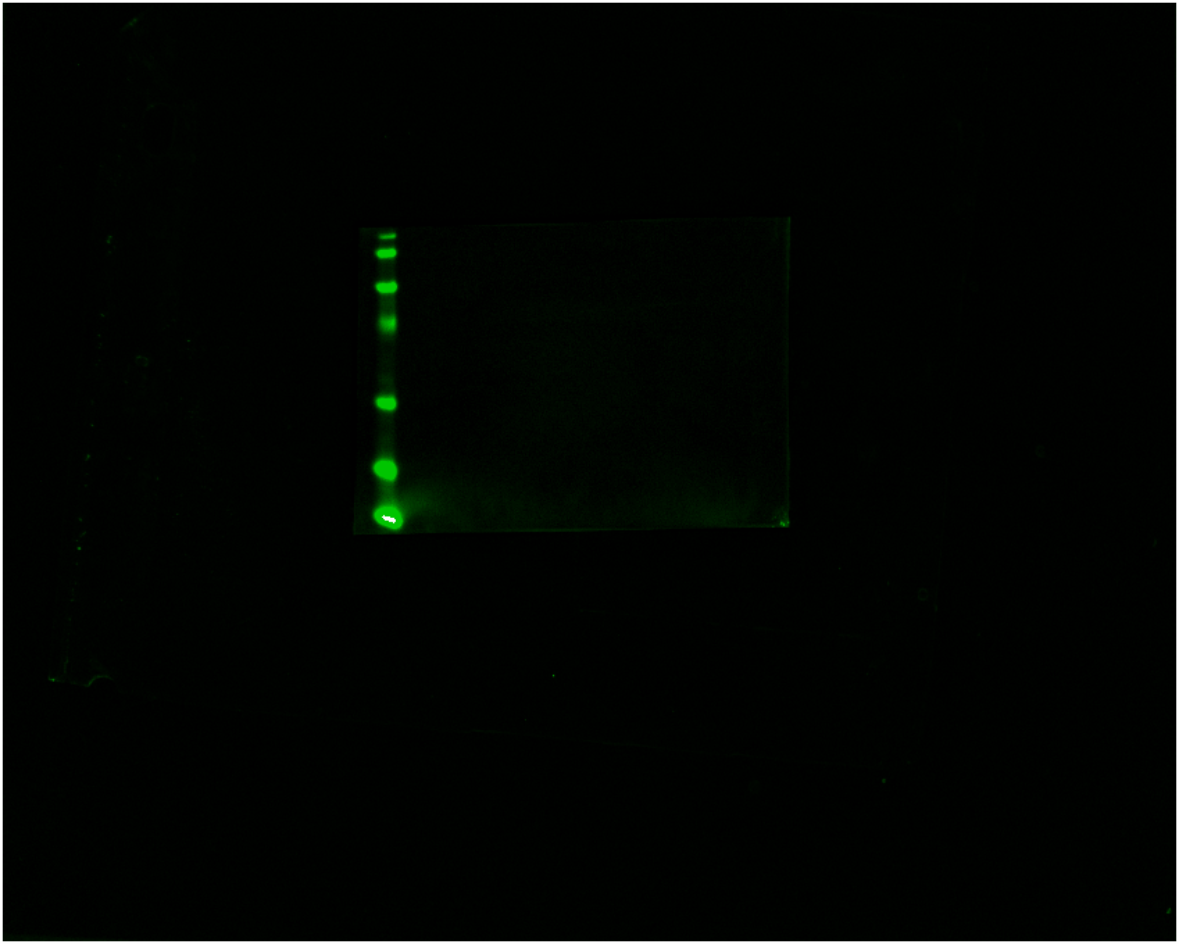

Supplement: Figure 1—figure supplement 1—source data 2. [file elife-107650-fig1-figsupp1-data2.zip › FigureS1d-original/UNIVERSAL_04112025_123514IRDye700.tif]

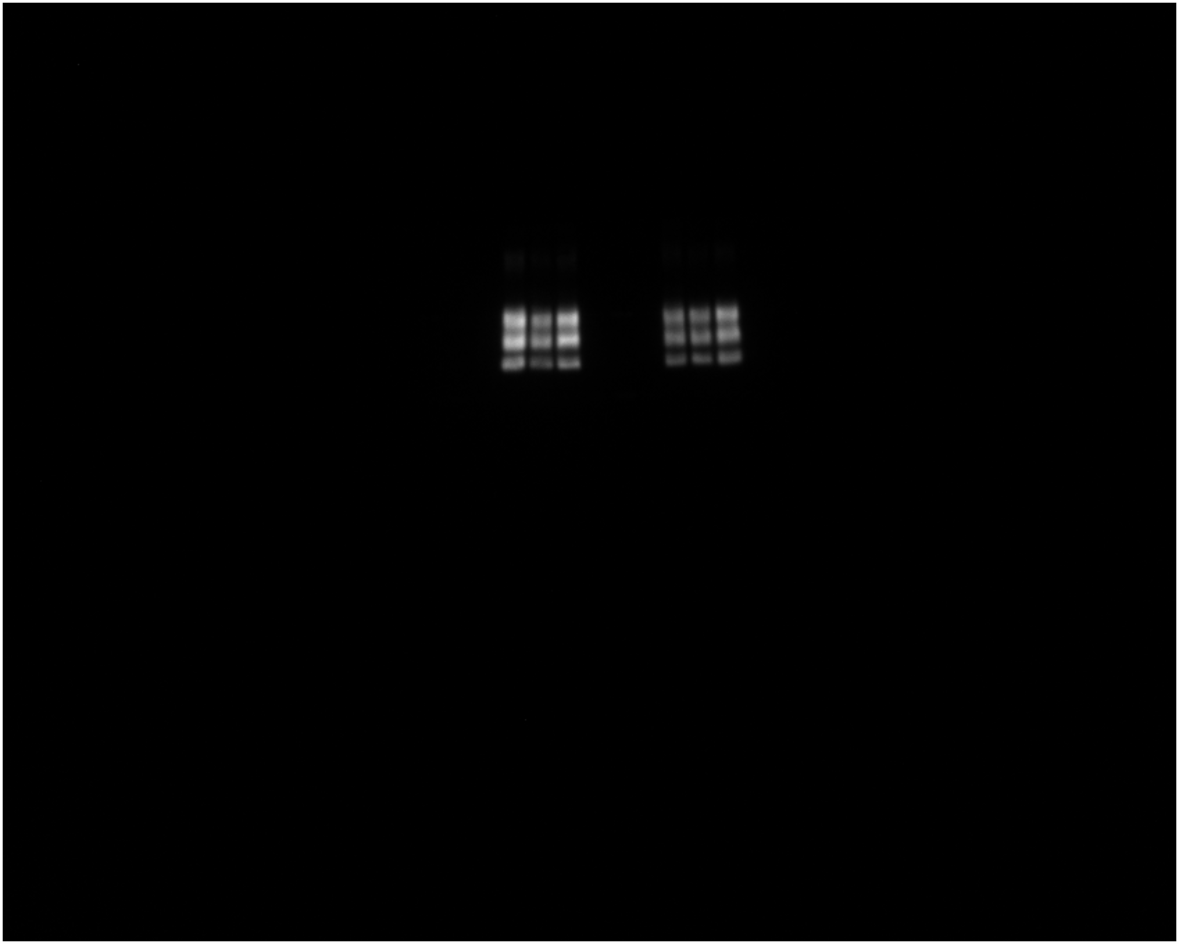

Supplement: Figure 1—figure supplement 1—source data 2. [file elife-107650-fig1-figsupp1-data2.zip › FigureS1d-original/UNIVERSAL_04112025_123514SuperSignalWestPicoPlus.tif]

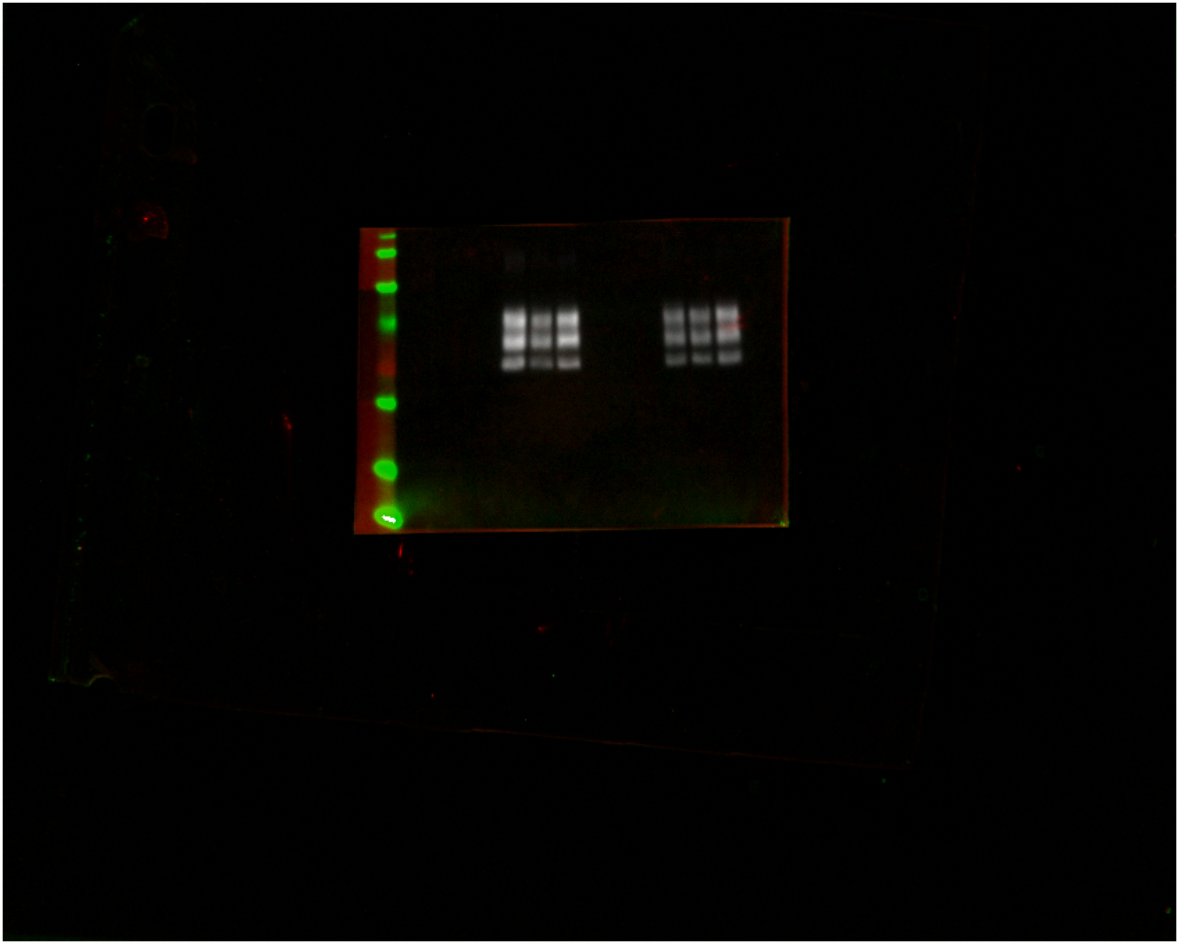

Supplement: Figure 1—figure supplement 1—source data 2. [file elife-107650-fig1-figsupp1-data2.zip › FigureS1d-original/UNIVERSAL_04112025_123514_composite.tif]

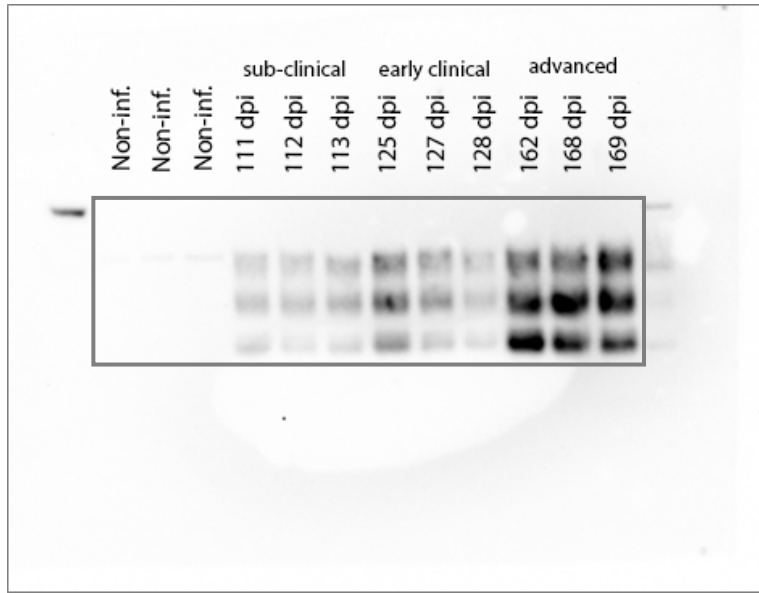

Supplement: Figure 2—figure supplement 1—source data 1. [file elife-107650-fig2-figsupp1-data1.zip › FigureS2a-labeled.pdf]

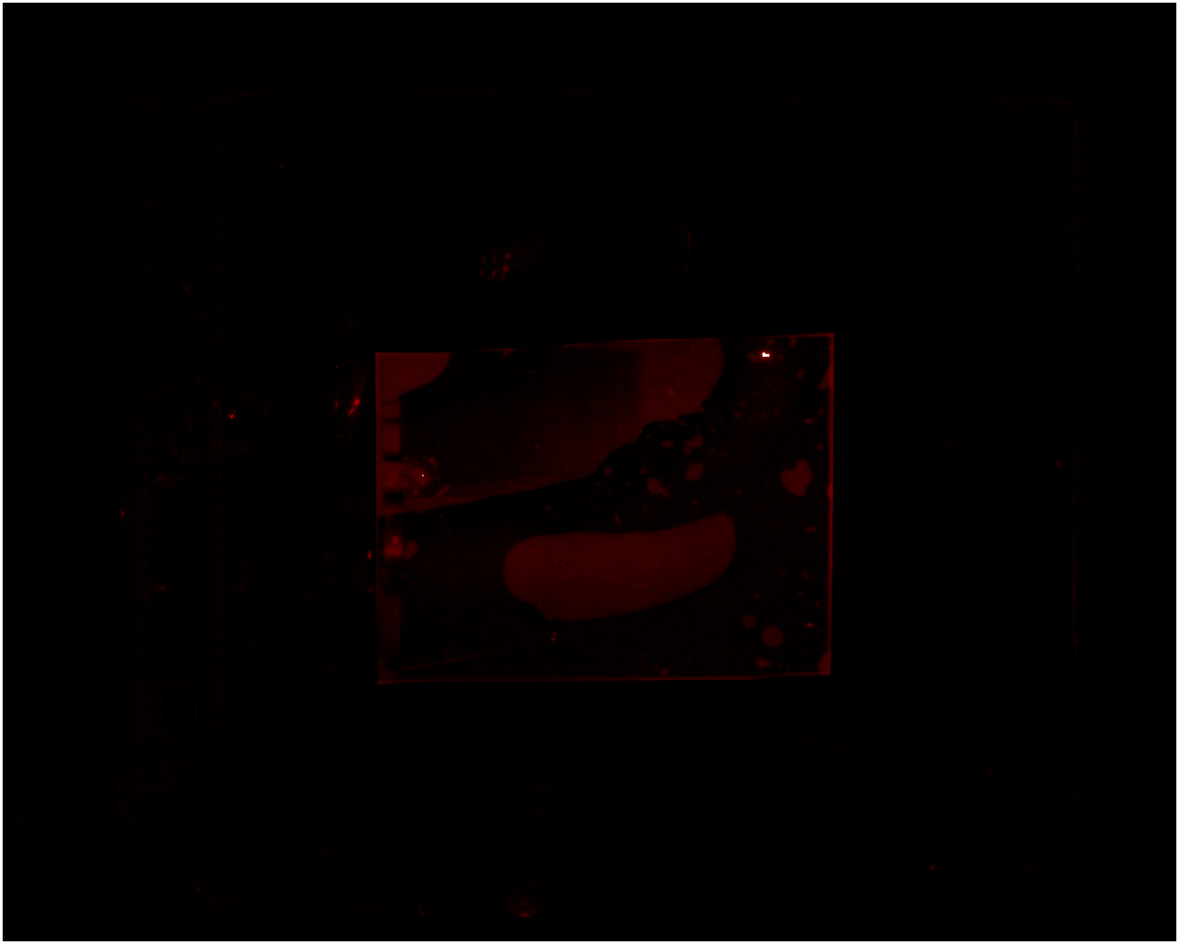

Supplement: Figure 2—figure supplement 1—source data 2. [file elife-107650-fig2-figsupp1-data2.zip › FigureS2a-original/UNIVERSAL_03132025_134540AlexaFluor546.tif]

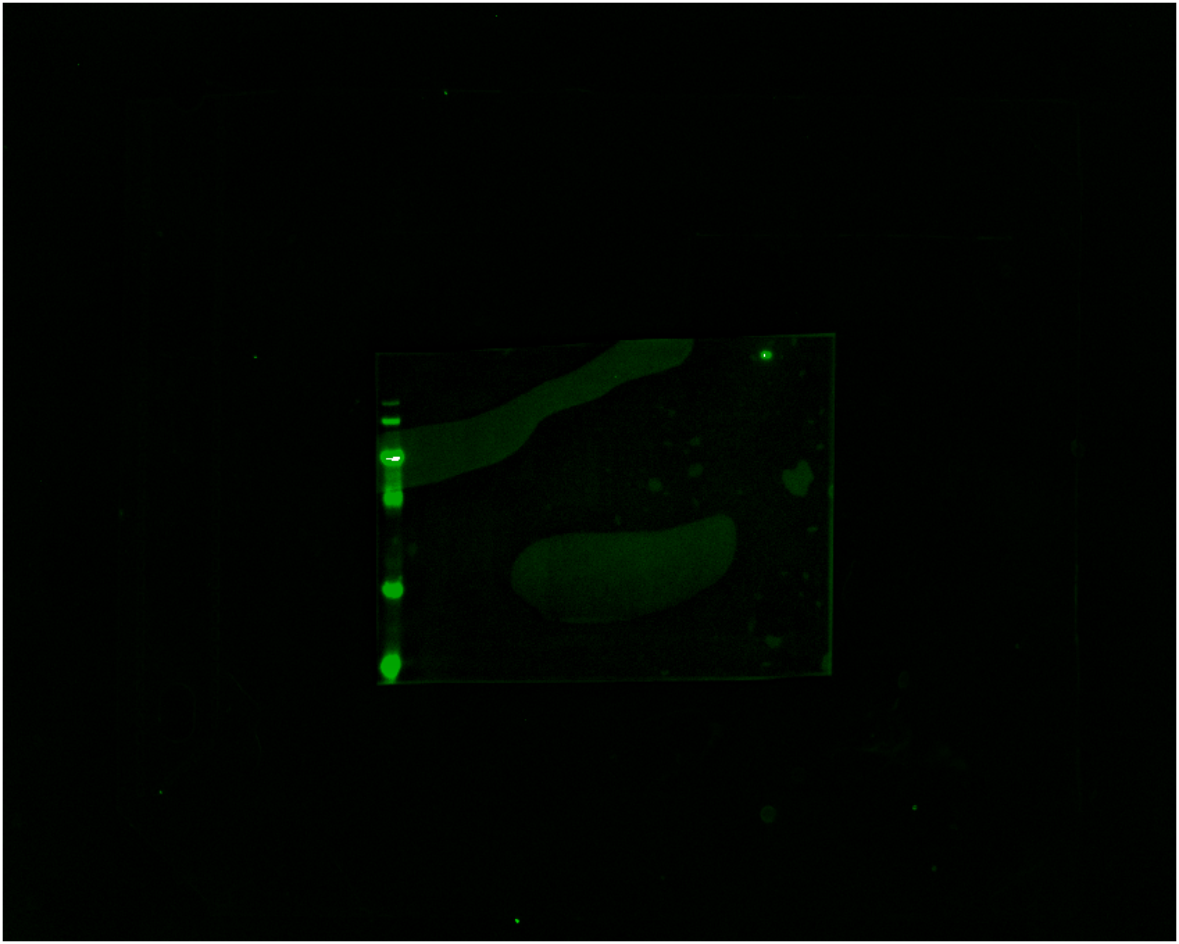

Supplement: Figure 2—figure supplement 1—source data 2. [file elife-107650-fig2-figsupp1-data2.zip › FigureS2a-original/UNIVERSAL_03132025_134540IRDye700.tif]

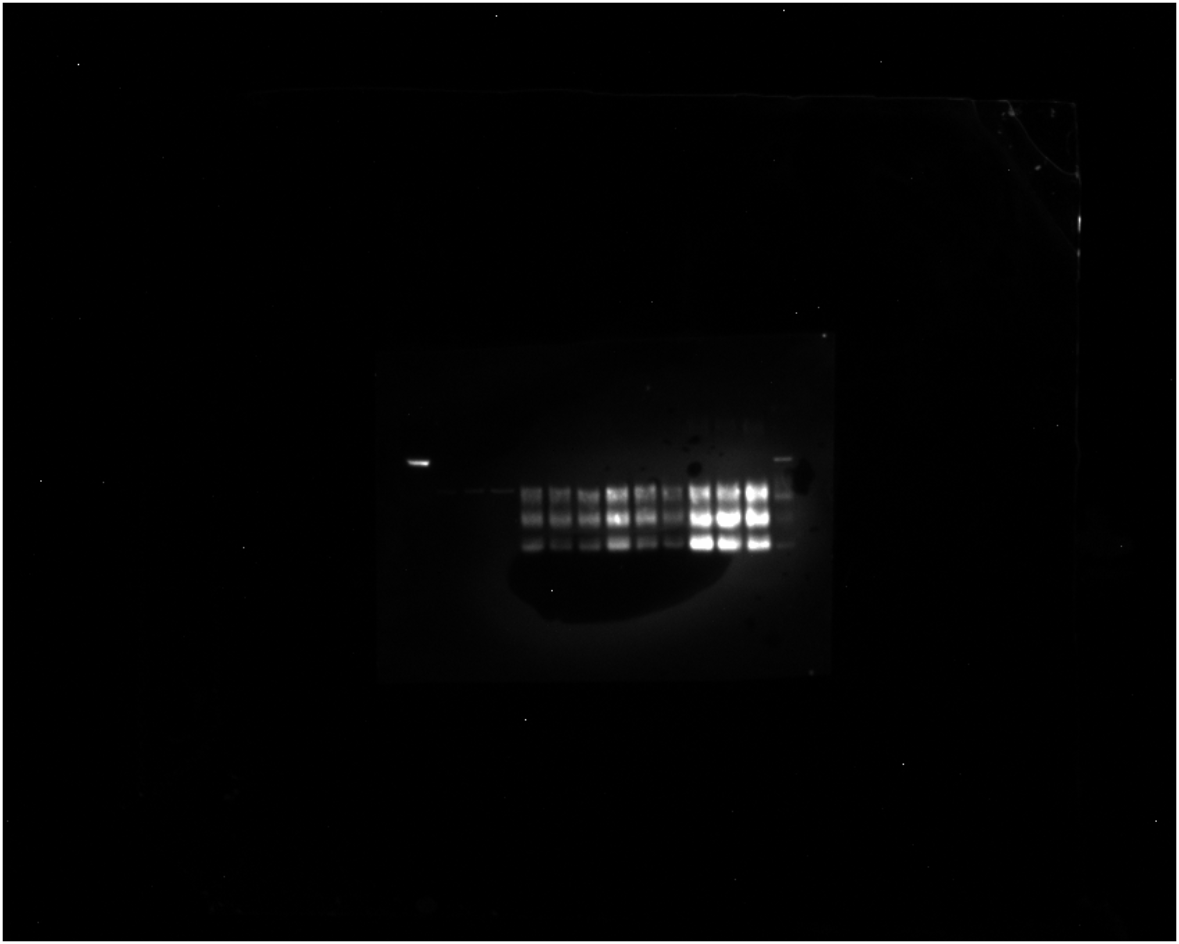

Supplement: Figure 2—figure supplement 1—source data 2. [file elife-107650-fig2-figsupp1-data2.zip › FigureS2a-original/UNIVERSAL_03132025_134540SuperSignalWestPicoPlus.tif]

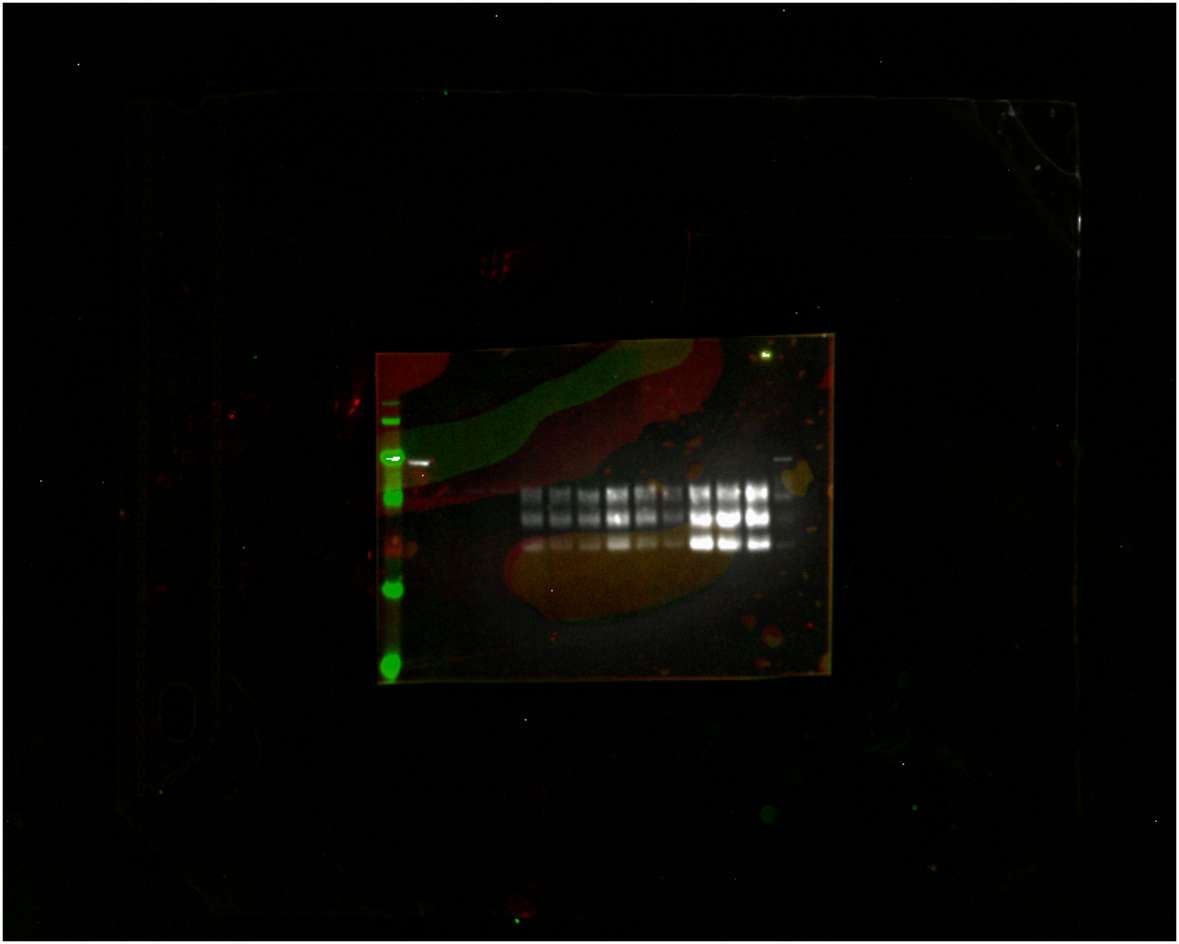

Supplement: Figure 2—figure supplement 1—source data 2. [file elife-107650-fig2-figsupp1-data2.zip › FigureS2a-original/UNIVERSAL_03132025_134540_composite.tif]
